# Semelparous Death as one Element of Iteroparous Aging Gone Large

**DOI:** 10.3389/fgene.2022.880343

**Published:** 2022-06-09

**Authors:** Carina C. Kern, David Gems

**Affiliations:** Institute of Healthy Ageing, Research Department of Genetics, Evolution and Environment, University College London, London, United Kingdom

**Keywords:** aging, biomass repurposing, *C. elegans*, insulin/IGF-1 signaling, programmatic aging, reproductive death, semelparity, senescent pathology

## Abstract

The aging process in semelparous and iteroparous species is different, but how different? Death in semelparous organisms (e.g., Pacific salmon) results from suicidal reproductive effort (reproductive death). Aging (senescence) in iteroparous organisms such as humans is often viewed as a quite different process. Recent findings suggest that the nematode *Caenorhabditis elegans*, widely used to study aging, undergoes reproductive death. In post-reproductive *C. elegans* hermaphrodites, intestinal biomass is repurposed to produce yolk which when vented serves as a milk to support larval growth. This apparent benefit of lactation comes at the cost of intestinal atrophy in the mother. Germline removal and inhibition of insulin/IGF-1 signaling (IIS) suppress *C. elegans* reproductive pathology and greatly increase lifespan. Blocking sexual maturity, e.g., by gonadectomy, suppresses reproductive death thereby strongly increasing lifespan in semelparous organisms, but typically has little effect on lifespan in iteroparous ones. Similarly, reduced IIS causes relatively modest increases in lifespan in iteroparous organisms. We argue that the more regulated and plastic mechanisms of senescence in semelparous organisms, involving costly resource reallocation under endocrine control, exist as one extreme of an etiological continuum with mechanisms operative in iteroparous organisms. We suggest that reproductive death evolved by exaggeration of mechanisms operative in iteroparous species, where other mechanisms also promote senescence. Thus, knowledge of *C. elegans* senescence can guide understanding of mechanisms contributing to human aging.

## Introduction

The study of the biology of aging takes place in two largely separate worlds. The first is that of biogerontology, where aging is studied in its entirety. The second is the much larger field of research on the many individual diseases of aging (such as cardiovascular disease, cancer, chronic obstructive pulmonary disease and Alzheimer’s disease). These two worlds are currently somewhat at odds with each other. After all, does it really make sense to study aging as a whole, given that diseases of aging are extremely diverse and vary greatly in terms of whether and when they present in different individuals? Can one in any meaningful sense define an aging process that underlies this disease multiplicity and variation? Is it not more plausible that many distinct diseases and pathologies develop in a time-dependent fashion (Peto and Doll, 1997)? Perhaps the strongest argument for studying aging as a whole is the existence of plasticity in overall aging rate. This manifests as evolutionary plasticity, evident from the large differences in lifespan between species (Finch, 1990a), and experimentally-induced alteration in aging rate in laboratory model organisms.

Aging rate varies greatly across species, from organisms in which aging is undetectable, such as the freshwater polyp *Hydra vulgaris* (Martínez, 1998; Schaible et al., 2015), and the sponge *Monorhaphis chuni* which can live at least 11,000 years (Jochum et al., 2012), to mayflies where adults die within days or even hours of eclosion. Particularly striking are the large differences seen between species that are anatomically similar. For example, free-living nematodes of the species *Caenorhabditis elegans* die of old age after only 2–3 weeks while the parasitic nematode *Loa loa* can live for up to 21 years (Klass, 1977; Richardson et al., 2012). Major differences are seen between even very closely related species, such as the common chimpanzee *Pan troglodytes* and our own species, with maximum lifespans of 68 and 122 years (Robine and Allard, 1998; Havercamp et al., 2019). In some cases single species possess different adult forms that age at different rates: in the nematode *Strongyloides ratti*, free-living adult females have a maximum lifespan of 5 days while karyotypically equivalent parasitic females can live for up to 403 days, an 80-fold difference in lifespan (Gardner et al., 2006). From this striking biodiversity in aging rate it can be deduced not only that aging rate is largely specified by the wild-type genome, but also that it can evolve rapidly. This aging process whose rate exhibits such a high degree of evolutionary plasticity includes the determinants of the panoply of diseases of aging.

The other kind of plasticity, that seen in laboratory model organisms in response to specific interventions, has been a major focus of experimental research in biogerontology. This includes two interventions in particular. Dietary (or caloric) restriction (DR) is the controlled reduction in food availability, discovered over a century ago to extend lifespan and improve late-life health in laboratory rodents (Osborne et al., 1917; Masoro, 2005). Studies of long-lived mutants led to the discovery of an endocrine and cellular signalling network which in the wild type shortens lifespan. This includes growth hormone (GH), the insulin/IGF-1 signalling (IIS) pathway and the target of rapamycin (mTOR) pathway (Kapahi et al., 2010; Kenyon, 2010; Bartke, 2019). An important objective of research on DR and GH/IIS/mTOR is to understand the proximate mechanisms of aging itself that such interventions act upon, and which remain poorly understood. This would help to understand etiologies of diseases of aging.

Research on the evolutionary (ultimate) and mechanistic (proximate) causes of aging are mutually informative, and development of an integrated, ultimate-proximate understanding is another major objective in research on aging. The well-established evolutionary theory of aging has informed the development of hypotheses about mechanistic theories of aging (Kirkwood and Holliday, 1979; Flatt and Schmidt, 2009; Maklakov and Chapman, 2019). Conversely, discovering determinants of aging in model organisms can provide understanding of the genes and cellular mechanisms involved in lifespan evolution, the so called evo-gero (evolutionary gerontology) approach (Partridge and Gems, 2006).

Here we discuss the possible mechanistic basis of the exceptional plasticity in aging seen in the nematode *C. elegans*, drawing on recent advances. This plasticity raises a number of questions relating to the comparative biology of aging. Could plasticity of this type exist in humans? Could intervention in it be achieved to protect against diseases of aging? What is the relationship between evolutionary plasticity in aging, and the lifespan plasticity observed in model organisms? And, can the mechanisms of lifespan plasticity in model organisms explain the evolution of species differences in lifespan? We describe how, after exploring a series of experimental blind alleys and inadequate hypotheses, we arrived at new, tentative answers to these questions. These draw in particular on the evolutionary relationship between aging in iteroparous and semelparous organisms.

## Identifying Proximate Mechanisms of Aging: Stochastic or Programmatic?

**FIGURE 1:**
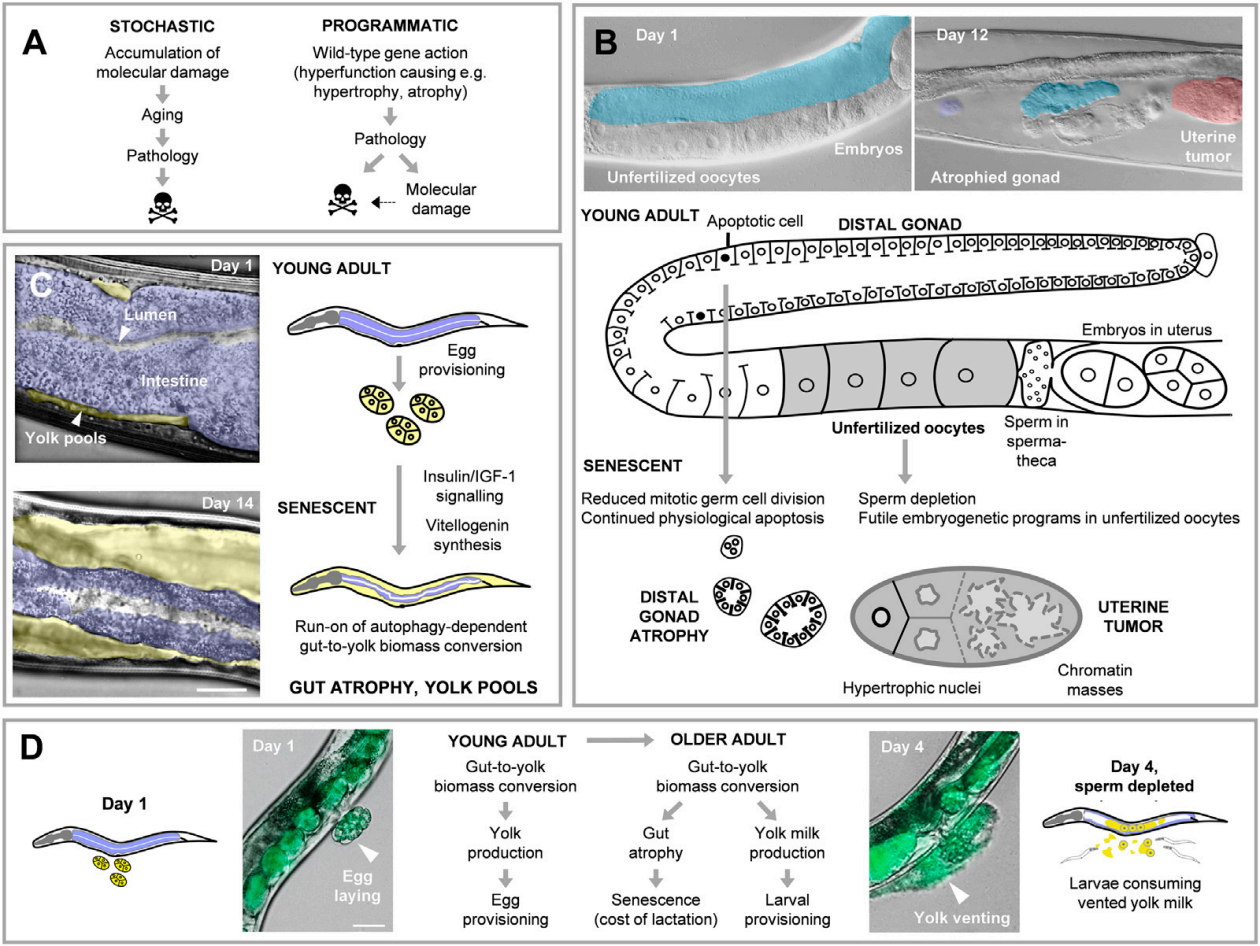
Programmatic mechanisms of aging are a cause of senescent pathology in *C. elegans*. **(A)** Stochastic and programmatic models of aging. These are not mutually exclusive, and are elements of a wider, multifactorial aging process (Gems, 2022). **(B)** Quasi-programs promote distal gonad atrophy and uterine tumor development. Futile run-on of physiological apoptosis promotes distal gonad atrophy and fragmentation (de la Guardia et al., 2016), along with depletion of the germline stem and progenitor cell pools (Tolkin and Hubbard, 2021). An embryogenetic quasi-program causes unfertilized oocytes to develop into teratoma-like uterine tumors (Wang et al., 2018). Blue, distal gonad. Red, uterine tumor. **(C)** Intestinal biomass repurposing supports yolk synthesis (vitellogenesis), whose seemingly futile run-on leads to intestinal atrophy (Ezcurra et al., 2018). Violet, intestine. Yellow, yolk pools in body cavity. **(D)** Intestinal atrophy as a cost of lactation. The recent demonstration that yolk is vented, and of promotion of larval growth by vented yolk, implies that later yolk production is not futile run-on, and that gut atrophy is a cost of lactation (Kern et al., 2021).

In *C. elegans*, single gene mutations reducing IIS can cause up to 10-fold increases in lifespan (Ayyadevara et al., 2008). Given the experimental tractability of *C. elegans*, the prospects for identifying the processes of aging itself through which IIS acts initially appeared very good. However, they have proven to be surprisingly difficult to discover. Initial attempts in the 1990s tested the then fashionable theory that stochastic (random) oxidative damage is the main cause of aging (Honda and Honda, 1999; Vanfleteren, 1993) (Figure 1A); however, it now appears unlikely that reactive oxygen species (ROS) are a major primary cause of aging, and certainly not in *C. elegans* (Gems and Doonan, 2009; Shields et al., 2021). Further attempts drew on a conception of aging as caused by a broad spectrum of chemicals causing diverse forms of molecular damage (Pletcher et al., 2007; Shore and Ruvkun, 2013). This included a focus on the phase 1, phase 2 biotransformation (drug metabolism) system which is upregulated in a number of long-lived mutants (Amador-Noguez et al., 2004; Gems and McElwee, 2005; Amador-Noguez et al., 2007; McElwee et al., 2007). However, although there is some evidence that drug metabolizing enzymes (DMEs) can promote *C. elegans* longevity (Ayyadevara et al., 2005a; Ayyadevara et al., 2005b), their importance in aging remains uncertain, particularly since upregulation of DMEs in long-lived mutants can in some cases be dissociated from longevity, in *C. elegans* (Guerrero et al., 2021) and *Drosophila* (Afschar et al., 2016). A third line of investigation pursued the possibility that aging is promoted by a progressive loss of protein folding homeostasis. While there is clear evidence that reduced IIS and reduced germline signaling can protect against such decline (Walker et al., 2001; Lithgow and Walker, 2002; Hsu et al., 2003; Labbadia and Morimoto, 2015) the mechanisms that cause such decline remain unclear, and appear more programmatic than stochastic (Ben-Zvi et al., 2009; Labbadia and Morimoto, 2014).

The apparent inadequacy of traditional, molecular damage theories to explain IIS effects on *C. elegans* led us to explore alternative, programmatic theories of aging (de Magalhães and Church, 2005; Blagosklonny, 2006; Gems and de la Guardia, 2013; Maklakov and Chapman, 2019), reviewed in Gems (2022). A starting point for such theories is the finding that many pathways found to influence aging (e.g., GH, IGF-1, mTOR) also promote growth and development. This suggests that senescent pathophysiology includes developmental processes. One possibility is that complex biological programs that promote fitness in early life run on in a futile fashion in later life, causing pathology (de Magalhães and Church, 2005; Blagosklonny, 2006). This hypothesis is in line with the evolutionary principle of antagonistic pleiotropy. Here natural selection can favor a new, pleiotropic allele which promotes early life fitness but causes later pathology, through late-life, harmful action of wild-type genes (Williams, 1957). This is because gene effects earlier in life have a greater impact on fitness (Medawar, 1952). According to the new theory, early life programs promoting fitness subsequently become futile quasi-programs; here the *quasi-*prefix refers to the fact that they are programmed in the mechanistic but not the adaptive sense (Blagosklonny, 2006). Thus, aging (or, at least, aging as promoted by growth pathways) is not programmed, but it can be said to be *programmatic* (Figure 1A).

There is evidence that quasi-programs contribute significantly to the development of multiple senescent pathologies in *C. elegans* (Gems and de la Guardia, 2013). These include gonad atrophy and fragmentation (de la Guardia et al., 2016), and uterine tumor development (Wang et al., 2018) (Figure 1B). One example of the mechanisms concerned involves yolk production in hermaphrodites; note that *C. elegans* reproduce largely as self-fertilizing hermaphrodites, and when sperm stocks are depleted on around day 4 of adulthood, reproduction ceases (Ward and Carrel, 1979). In post-reproductive hermaphrodites, yolk production by the intestine continues in post-reproductive adults in a seemingly futile fashion (Herndon et al., 2002; Depina et al., 2011). Such yolk production is provisioned by autophagy-mediated consumption of intestinal biomass, which leads to intestinal atrophy and formation of oily yolk pools in the body cavity (Garigan et al., 2002; Ezcurra et al., 2018; Sornda et al., 2019). This process is promoted by IIS, which activates vitellogenin (yolk protein) synthesis, and is suppressed in long-lived IIS mutants (Murphy et al., 2003; Depina et al., 2011; Ezcurra et al., 2018) (Figure 1C).

## Nematode Lactation: A Costly Program

For a while, we believed that we might in some sense have gotten to the bottom of things: wild-type IIS appeared to accelerate aging, at least in part, by promoting a vitellogenic quasi-program. However, our more recent findings imply that this was still not correct. We recently described how post-reproductive *C. elegans* vent yolk through the vulva, and that such yolk can be consumed by larvae, promoting their growth (Kern et al., 2021). This implies that vented yolk serves a function similar to milk (*yolk milk*), though whether this behavior really promotes fitness in the wild remains to be demonstrated. Milk feeding is occasionally seen in invertebrates, as in the Pacific beetle cockroach and the tsetse fly (Marchal et al., 2013; Benoit et al., 2015). Thus, post-reproductive yolk synthesis is not futile after all. What IIS is promoting is not a futile vitellogenic quasi-program but a fitness-promoting lactational program. The oily, vitellogenin-rich pools accumulating in older mothers are not a steatotic pathology but depots of yolk milk awaiting delivery to the hungry mouths of larvae. The intestinal atrophy is not the consequence of a quasi-program, but instead a cost of lactation (Figure 1D).

Based on these results, one can elaborate upon and extend the existing programmatic theory of aging. First, these findings do not argue against the role of quasi-programs in *C. elegans* aging. The development of teratoma-like uterine tumors is, seemingly, a consequence of action of embryogenetic quasi-programs in unfertilized oocytes (Wang et al., 2018). By contrast, intestinal atrophy is promoted by a different class of pathogenic program, which have been dubbed *costly programs* (Gems et al., 2021).

The programmatic theory emphasizes the importance in aging of gene *action* as opposed to the *passive* process of damage accumulation - what has been referred to as hyperfunction (Blagosklonny, 2008). We previously proposed that post-reproductive yolk production represents a quasi-program resulting from IIS hyperfunction (Ezcurra et al., 2018). But if instead a costly lactational program is operative, does that rule out a role for hyperfunction? We argue not, but instead that the lactational program is hyperfunctional with respect to intestinal health but not yolk milk production (Gems et al., 2021). According to this account, in costly programs, hyperfunction exists with respect to the organ that suffers the cost (Figures 1D, 3A).

## *C. elegans* is Semelparous: Reproductive Death and Adaptive Death

How likely is it that costly programs as exemplified by *C. elegans* lactation contribute to the pathophysiology of diseases of iteroparous organisms such as humans? Atrophy of multiple organs due to costly programs involving bulk biomass repurposing is not typical of mammalian aging. However, it often occurs in species of animals and plants that exhibit suicidal reproductive effort. Based on their life history, metazoa can be approximately divided into iteroparous species with the capacity for multiple rounds of reproduction, and semelparous species that reproduce once. Semelparous organisms exhibiting terminal reproductive effort leading to death (or *reproductive death*) include a few vertebrates, mainly among fish, such as Pacific salmon, lampreys and eels, and many plants (monocarpy) (Finch, 1990b). Such terminal reproductive effort often involves somatic biomass repurposing, where tissues and organs are consumed to provide material for reproductive effort (Gems et al., 2021). For example, in Pacific salmon body wall muscle is consumed for energy for swimming up river and growth of secondary sexual characteristics such as the hump and beak (Robertson et al., 1961; Finch, 1990b; Quinn and Foote, 1994). In many monocarpic plants somatic tissues throughout the organism are consumed to provide resources for seeds; for example, 90% of nitrogen in rice grains are derived from breakdown of somatic tissues (Diaz-Mendoza et al., 2016).

This raises the possibility that *C. elegans* is a semelparous species, undergoing reproductive death. In fact, many features of *C. elegans* aging are consistent with this view. For example, in iteroparous species such as humans, aging-related diseases accumulate towards the end of life after an extended period of healthy adulthood. By contrast, in adult *C. elegans* hermaphrodites senescent decline begins within hours of sexual maturity (Labbadia and Morimoto, 2015), and major pathological changes in internal anatomy develop in relative synchrony (Garigan et al., 2002; McGee et al., 2011; McGee et al., 2012; de la Guardia et al., 2016; Ezcurra et al., 2018; Wang et al., 2018). Reproductive death is traditionally viewed as a phenomenon distinct from aging as seen in iteroparous species, as is the clearly programmatic process of leaf senescence in deciduous plants (Finch, 1990b).

**FIGURE 2:**
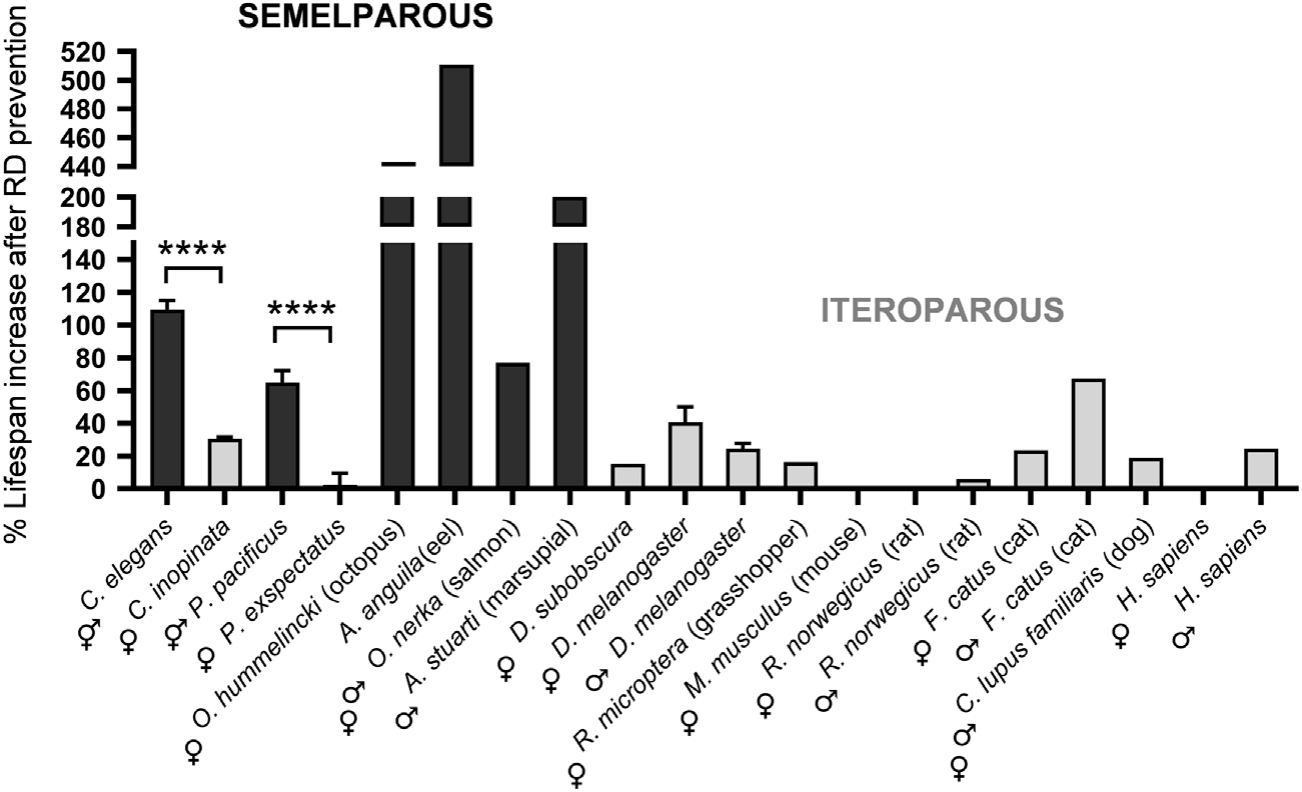
Blocking reproductive death in semelparous species strongly increases lifespan, while gonadectomy in iteroparous species overall does not. Effects on lifespan of germline ablation in hermaphrodite but not female nematodes of several species resemble effects of blocking reproductive death in semelparous species (Kern et al., 2021). Values for nematodes from a recent report (Kern et al., 2021), and for other species from a recent review (Gems et al., 2021); where there are multiple studies of the same species, only references with both male and female data are included, and for *A. anguila* (eel) data from the more recent source (Tesch, 1977).

If reproductive death is blocked by gonadectomy or behavioral manipulation there results, unsurprisingly, a large increase in lifespan. This has been demonstrated in a range of semelparous species, both animals and plants (Gems et al., 2021) (Figure 2). For example, gonadectomy of Pacific salmon prevents reproductive death, leading to extension of lifespan of up to 3.7 years (+77%) (Robertson, 1961). This suggests a possible parallel with the effects of germline ablation in *C. elegans*, which causes a large increase lifespan (+64%) (Hsin and Kenyon, 1999) and suppresses senescent pathology, including intestinal atrophy (Ezcurra et al., 2018). Does germline ablation increase *C. elegans* lifespan by suppressing reproductive death?

We recently explored this by a comparative analysis of *Caenorhabditis* species. We reasoned that lactation might have evolved in *C. elegans* as a means to overcome the inbuilt reproductive redundancy caused by protandrous hermaphroditism (the *C. elegans* hermaphrodite germline produces first sperm and then oocytes, which limits self-sperm number and therefore reproductive span). Somatic biomass repurposing to support yolk milk production allows post-reproductive hermaphrodites to continue to express fitness benefits. This predicts that *Caenorhabditis* females will neither lactate or show lactation-associated pathologies. Comparison of three *Caenorhabditis* sibling species pairs, each with one androdioecious (hermaphrodites, males) and one gonochoristic (females, males) species showed lactation in hermaphrodites but not females in each case. Importantly, germline ablation strongly increased hermaphrodite lifespan but not female lifespan in all three species pairs (Kern et al., 2020) (Figure 2). This supports the view that germline ablation increases *C. elegans* lifespan by suppressing reproductive death.

It is also plausible that an additional form of programmatic life-shorting mechanism is operative in *C. elegans*: adaptive death. This has been reviewed previously (Galimov et al., 2019; Lohr et al., 2019) but, briefly, adaptive death is a relatively rare phenomenon restricted to species with a colonial lifestyle (viscous populations of closely related individuals), where colony level selection can occur. Here death of individuals can increase colony fitness in a manner analogous to action of apoptosis of cells within multicellular organisms. Evolutionary theory predicts that adaptive death can more readily evolve in semelparous organisms because evolved mechanisms that hasten death after reproduction will not reduce individual fitness. For example, there is evidence that adaptive death occurs in Pacific salmon, where nutrients from carcasses support growth of fish fry (Cederholm et al., 1999; Galimov and Gems, 2021).

## Good News: AN Iteroparity Semelparity Continuum

The occurrence of lactation coupled to reproductive death and adaptive death in *C. elegans* provide a potential explanation for the evolved, highly life-shortening effects of the wild-type germline and wild-type IIS, which have remained an enigma since their discovery (Partridge and Harvey, 1993). However, if *C. elegans* lifespan is a function of reproductive death but human lifespan is not, and if reproductive death is a process distinct from aging then *C. elegans* is, on the face of it, an unhappy choice as a model organism to study aging. Do these findings sound the death knell for *C. elegans* aging research? Is this a disaster for biogerontology? We believe not. However, our conclusions, assuming they are correct, do warrant a shift in understanding of *C. elegans* lifespan and its relevance to aging in iteroparous species, in two respects, one bad and one good.

First the bad news. The extraordinary finding some 30 years ago that single gene mutations can greatly increase *C. elegans* lifespan (Friedman and Johnson, 1988; Kenyon et al., 1993) suggested that some central aging process exists that determines all late-life disease and is amenable to major intervention. The mystery of this process in *C. elegans* has inspired generations of biogerontologists. Our conclusions, if correct, demystify this effect, and explain it away as suppression of reproductive death, akin to that in castrated salmon. The implied prospect of applying knowledge from *C. elegans* to radically extend human lifespan consequently fades, dispelled like a desert mirage.

Next, a more positive perspective. Wild-type growth control pathways do shorten lifespan in iteroparous organisms, although to a much smaller degree (in relative terms) to *C. elegans* (Ayyadevara et al., 2008; Slack et al., 2011; Foukas et al., 2013). This could imply that mechanisms that cause rapid death in semelparous organisms are a minor contributory factor in iteroparous aging. We have postulated that mechanisms of reproductive death evolve by amplification of costly reproductive programs that are present in iteroparous organisms. However, in iteroparous organisms, such programmatic etiologies may act in concert with other classes of senescent etiology (Gems et al., 2021). Similar neuroendocrine and steroid hormone signaling networks control growth, reproduction and lifespan in semelparous and iteroparous organisms (Gems et al., 2021), consistent with the costly program amplification hypothesis; whether amplified GH/IIS/mTOR signalling in particular drives reproductive death in semelparous higher animals (e.g., Pacific salmon, marsupial mice) remains unexplored.

**FIGURE 3:**
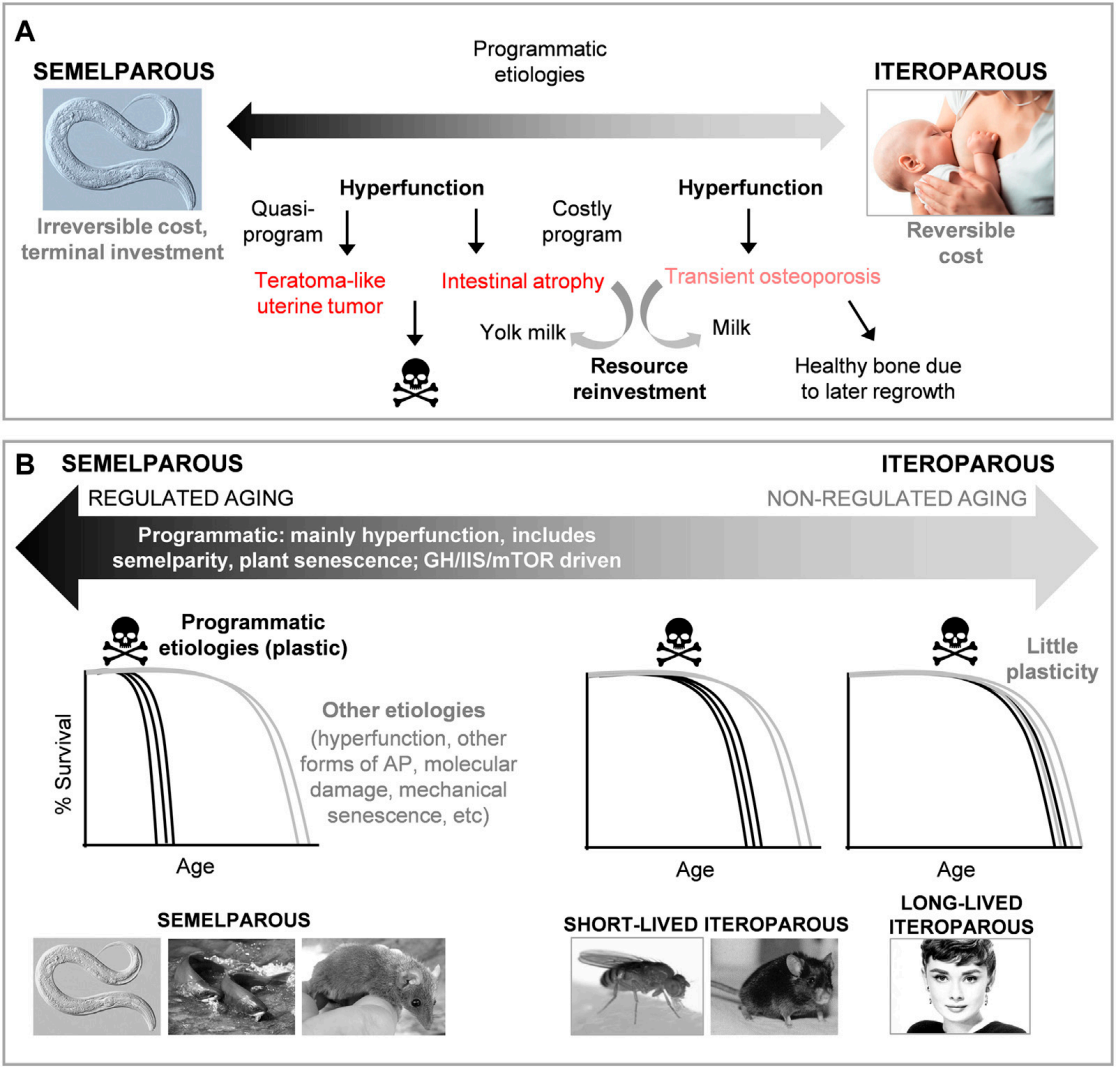
The relationship between aging in semelparous and iteroparous organisms. **(A)** Aging in semelparous and iteroparous organisms lie on a single etiological spectrum. Semelparity evolves from iteroparity, including amplification of costly programs present in the latter. These change from being mild and reversible, to severe and terminal. **(B)** Regulated vs. non-regulated aging. Levels of phenotypic plasticity in aging vary greatly between organisms, being greatest in semelparous organisms, and smallest in long-lived iteroparous organisms. The plastic component of aging is regulated and highly programmatic. If such regulated aging is suppressed, more diverse mechanisms of aging may become life limiting, including other modes of antagonistic pleiotropy (AP), stochastic molecular damage (e.g., DNA damage), and mechanical senescence. Semelparous organisms shown are *C. elegans*, Pacific salmon (*Oncorhynchus nerka*), and the brown antechinus (*Antechinus stuartii*), a marsupial.

Moreover, while in semelparous organisms such costs are terminal, promoting death, in iteroparous organisms they are often reversible. For example, during mammalian lactation consumption of bone occurs to effect calcium repurposing for milk production (Speakman, 2008). This leads to transient osteoporosis which is reversed after lactation by bone regrowth. By contrast, intestinal atrophy coupled to yolk milk production in *C. elegans* is irreversible (Figure 3A). This suggests that effects of growth control pathways (GH/IIS/mTOR) on aging may act to a large extent through programmatic mechanisms (e.g., costly programs, quasi-programs) which constitute one element of the aging process, and one that is particularly plastic. According to this view, semelparous organisms (such as *C. elegans*) die from a subset of mechanisms of iteroparous aging that have grown large. This makes them a convenient model for studying one component of the intractably multifactorial process of iteroparous aging. Thus, aging in semelparous and iteroparous organisms are not wholly different processes; this is consistent with the observed cross-species continuum between semelparity and iteroparity (Hughes, 2017).

## Regulated vs. Non-Regulated Aging

The semelparity-iteroparity continuum model has further consequences in terms of reconceiving the nature of aging. If *C. elegans*, a semelparous organism in which aging is highly programmatic, is a good model organism for learning about human aging, then so are other semelparous organisms, including monocarpic plants. Mechanisms of biomass repurposing coupled to somatic senescence are particularly well characterised in plants (Young and Augspurger, 1991; Davies and Gan, 2012; Avila-Ospina et al., 2014). Arguably, more experts on plant senescence should be invited to biogerontology conferences.

It also suggests a new perspective on the significance of phenotypic plasticity in aging. In Robertson’s classic salmon gonadectomy study, reproductive death was replaced by senescence of a kind more typical of iteroparous species (though involving some similar pathological changes) (Robertson and Wexler, 1962). This illustrates a general feature of senescence: if one life-limiting cause of senescence is alleviated (e.g., hypertension leading to cardiovascular disease) another will take its place (e.g., cancer or dementia). It has been argued that growth control pathways (GH/IIS/mTOR) exert effects on lifespan largely through programmatic mechanisms (de Magalhães and Church, 2005; Blagosklonny, 2006), in both semelparous and iteroparous organisms (Gems et al., 2021). This suggests that across the semelparity-iteroparity spectrum one can distinguish two distinct elements of aging: that which is *regulated* and plastic, and that which is *non-regulated* and non-plastic (Figure 3B). Plausibly, non-regulated aging is caused by various broad classes of etiology, including those previously proposed: molecular damage (particularly DNA damage), mechanical senescence and, particularly, diverse modes of wild-type gene action, including AP of many genes with small effects (Figure 3B) (Williams, 1957; Vijg and Suh, 2013; Gems, 2022).

## Perspectives

Biogerontology lacks the broad, shared understanding (paradigms) of more mature fields of science such as chemistry or genetics (Gems and de Magalhães, 2021). Here we have groped towards a general understanding from the parochial perspective of *C. elegans* aging biology. We opened our essay by emphasizing the importance to biogerontology as a field of the existence of plasticity in the aging process as a whole. But then we end by arguing that regulated phenotypic plasticity in lifespan reflects one element of aging rather than senescence in its entirety.

In terms of the evolution of diversity in aging rate, we have presented an evo-gero type account, in which programmatic mechanisms in iteroparous species, for example costly programs, evolve into more destructive, life-shortening forms. We have suggested that something like this has occurred in the evolution of hermaphroditism in the nematode genus *Caenorhabditis* (and also *Pristionchus*) (Kern et al., 2020). However, our reasoning that such phenotypic plasticity does not involve the whole aging process suggests that mechanisms of regulated aging play only a small role in the much greater evolutionary plasticity in aging. In these arguments we can see the evo-gero approach (Partridge and Gems, 2006) contributing to our understanding of the evolution of aging.

Of considerable interest is the natural diversity in regulated aging, and the extent to which regulated aging occurs in higher animals, particularly humans. In discussing the latter, it is helpful to distinguish two different forms of plasticity in aging. First, *positive plasticity*, that which leads to decelerated aging and increased lifespan, relative to the wild type on a controlled diet that is optimal for fertility. Second, *negative plasticity*, that which accelerates aging, as in the response to high food intake (e.g., leading to harmfully high BMI in humans) (Blagosklonny, 2011; Horvath et al., 2014; Aune et al., 2016; Salvestrini et al., 2019). Negative plasticity is distinct from life-shortening effects of toxic food components, though distinguishing the two can be difficult (Piper and Partridge, 2007).

In rodents, mutant and dietary restriction studies have shown the existence of both positive and negative plasticity. By contrast, in humans, while aging can be strongly accelerated, as in individuals with progeroid syndromes and with high BMI, at most only modest deceleration has been detected (Zhang et al., 2020). No markedly long-lived human mutants have ever been found; it now seems possible that one uniquely long-lived individual, Jeanne Calment (died aged 122) was an imposter (her daughter Yvonne, who might have switched identity with her late mother to avoid inheritance tax) (Zak, 2019; Collins, 2020). GH receptor-defective mice show striking increases in lifespan (Coschigano et al., 2000), but the same defect in humans does not extend lifespan, though it does provide some health benefits (Guevara-Aguirre et al., 2011; Aguiar-Oliveira and Bartke, 2019). While DR is clearly beneficial in reducing harmfully high BMI, results of studies in both humans and rhesus macaques have not clearly demonstrated positive plasticity effects (Mattison et al., 2012; Mattison et al., 2017; Most et al., 2017). A marked extension of lifespan by DR has been seen in one primate, the mouse lemur (Pifferi et al., 2018), which may reflect either positive plasticity or a toxic food rescue effect. Thus, arguably, at present the best prospects for applying our understanding of mechanisms of plasticity to benefit human health are to combat the harmful effects of negative plasticity, which are a major cause of late-life disease (Blagosklonny, 2011; Aune et al., 2016).

In conclusion, the new ideas and findings described in this essay suggest that the prospect of applying the magic of *C. elegans* plasticity in aging to dramatically decelerate human aging was, regrettably, just a beautiful dream. This arose due to the false assumption that *C. elegans* lifespan is a function of a universal aging process, rather than of semelparous reproductive and adaptive death (Lohr et al., 2019; Gems et al., 2021). But the new perspectives provided should help us to wake up to a possibility that is just as exciting: that of a mature understanding of the aging process, and the deep insights into the causes of diseases of aging that it will bring.

## Data Availability

The original contributions presented in the study are included in the article, further inquiries can be directed to the corresponding author.
